# USING REFLECTIVE WRITING TO PROMOTE RESILIENCE

**DOI:** 10.1093/geroni/igz038.852

**Published:** 2019-11-08

**Authors:** Ana L Leech, Anson J Koshy

**Affiliations:** UT Houston McGovern Medical School, Houston, Texas, United States

## Abstract

Patients invite us, rather reluctantly sometimes, into their lives during some of their most difficult moments; these encounters change us even if it is not readily apparent. Gathering for this writing group, we have found that creating a permanent record of our deepest thoughts and feelings enables further analysis and discussion in a collegial and supportive environment. In this paper, I relate one clinical story as an example. I will never forget caring for Mr. R. during what I can only call an ordeal, but writing it down, exploring my chosen words, and sharing them with the team brought validation and closure to what would have been a deep wound in my soul. The scar remains, being remodeled by time, experience, and other wounds, softened through writing and sharing, becoming part of me. We explore one way in which supportive writing groups can foster professional relationships and growth.

